# Transcriptome Analysis of Ivosidenib-Mediated Inhibitory Functions on Non-Small Cell Lung Cancer

**DOI:** 10.3389/fonc.2021.626605

**Published:** 2021-03-30

**Authors:** Juan Wu, Ru Chen, Huiqing Shen, Ting Yan, Yu Qian, Yaping Zhang, Zhuoya Huang, Pengzhou Kong, Min Pang, Xinri Zhang

**Affiliations:** ^1^ Department of Respiratory and Critical Care Medicine, The First Hospital, Shanxi Medical University, Taiyuan, China; ^2^ Department of Pathology & Shanxi Key Laboratory of Carcinogenesis and Translational Research on Esophageal Cancer, Shanxi Medical University, Taiyuan, China

**Keywords:** ivosidenib, RNA-seq, bioinformatic analysis, ceRNA, non-small cell lung cancer

## Abstract

Ivosidenib is an isocitrate dehydrogenase mutant inhibitor that the US Food and Drug Administration recently approved for the treatment of leukemia. Studies suggested that ivosidenib may inhibit the progression of non-small cell lung cancer (NSCLC). In the present study, we explored RNAs and their potential regulatory mechanisms by which ivosidenib treats NSCLC cells. We used MTT assays, Transwell assays, and flow cytometry to measure the anti-tumor effects of ivosidenib in NSCLC cells. We performed whole transcriptome sequencing to determine differentially expressed mRNAs (DE-mRNAs) and non-coding RNAs (ncRNA). We used GO and KEGG pathway enrichment analyses to identify the functions and potential mechanisms. According to miRNA target interactions, we constructed a competing endogenous network. Ivosidenib inhibited the proliferation, invasion, and migration of NSCLC cells and inhibited tumor growth *in vivo*. We identified 212 DE-mRNAs, four DE-miRNAs, and 206 DE-lncRNAs in ivosidenib-treated NSCLC cells compared to untreated NSCLC cells. DE-mRNAs were significantly enriched in the cancer-associated pathways, including the TGF-β signaling pathway, the PI3K-Akt signaling pathway, the Jak-STAT signaling pathway, the MAPK signaling pathway, the Rap1 signaling pathway, and cell adhesion molecules. Based on the competing endogenous RNA hypothesis, we constructed lncRNA-miRNA-mRNA networks to elucidate the regulatory relationships between mRNA and ncRNA. We found that qRT-PCR results showed corresponding expression trends of differential genes with sequencing data. Our results provide insights into the molecular basis of ivosidenib suppression of NSCLC.

## Introduction

Lung carcinoma carries the highest incidence and mortality among cancers (1); we divide it into small cell and non-small cell lung cancer (NSCLC). Approximately 85% of lung carcinoma is NSCLC, which includes lung adenocarcinoma (40% of NSCLC), lung squamous cell carcinoma (40% of NSCLC), large cell carcinoma (10% of NSCLC), and other less common subtypes. Despite substantial progress in cancer therapies, the 5-year survival rate of NSCLC remained about 18%, suggesting an urgent need for new agents to combat this malignancy (2).

Isocitrate dehydrogenases participate in various aspects of cellular metabolism. Isocitrate dehydrogenase 1 (IDH1) converts isocitrate to α-ketoglutarate (α-KG) by reducing NADP+ to NADPH; this is important for reduction-oxidation balance that the glutathione and thioredoxin systems establish (3). Numerous studies showed mutations in IDH1 in several malignancies, the most common mutation being IDH1 R132H (4–6). IDH1 mutations (mIDH1) lead to abnormal IDH1 function that converts α-KG into 2-hydroxyglutarate (2-HG). MIDH1 occurs in low-grade (grade I and II) glioma (5), acute myeloid leukemia (AML) (6), chondrosarcoma (7), and T cell lymphomas (8). In a large population study, a total of 298 lung carcinoma samples [179 samples by Kang et al. (9), 107 samples by Bleeker et al. (10), and 12 samples by Tan et al. (11)] showed no IDH1 mutations. Rodriguez et al. analyzed IDH1/2 mutations in 1924 NSCLC specimens (92% adenocarcinoma) using next-generation sequencing and identified IDH1/2 mutations in nine (0.5%) adenocarcinomas (12). These findings suggest that mIDH1 in NSCLC is relatively rare. A large population-based study convincingly showed elevated levels of IDH1 transcription and translation in NSCLC tissues compared with those of paired normal tissues (11, 13). Owing to its favorable specificity and sensitivity, the IDH1 level may be a diagnostic marker for NSCLC diagnosis (11). A study showed that knockdown of IDH1 by RNA interference reduced the proliferative capacity of NSCLC cells and significantly decreased *in vivo* xenograft tumor formation, suggesting that IDH1 may be a potential target in lung cancer (11).

Ivosidenib (AG-120) is a potent inhibitor of the mIDH1 that has clinical activity and safety profiles. In 2018, investigators began using ivosidenib to treat leukemia (14). Studies showed that ivosidenib exhibited rapid-equilibrium inhibition against the mIDH-R132 homodimer; research also showed that ivosidenib bound and inhibited the IDH1-WT homodimer (14). These observations suggest that ivosidenib may inhibit the progression of other cancers with high IDH1 expression. In other words, ivosidenib may be a potential therapeutic drug candidate for NSCLC treatment. Nevertheless, its effects on NSCLC, as well as the potential mechanisms, remain unclear.

Long non-coding RNAs (lncRNAs) are ncRNAs that are 200 nucleotides in length. They regulate the expression of target genes transcriptionally and post-transcriptionally, without protein-coding function. A substantial body of evidence supports the involvement of lncRNAs in carcinogenesis and cancer progression (15). MiRNAs are endogenous non-coding small RNAs (ncRNAs) with 22 nucleotides that bind to 3’-UTR of target genes’ mRNA and negatively regulate their expression by inhibition of translation or degradation of mRNAs (16). Several lines of evidence suggest that miRNA mediates an extensive range of cancer processes, including cell proliferation, migration, invasion, and apoptosis (17). Fang et al. demonstrated that the overexpression of miR-20a-5p stimulated NSCLC to proliferate and invade (18). Zhang et al. suggested that miR-493-5p suppressed tumors in osteosarcoma cells; overexpression of miR-493-5p suppressed proliferation and metastasis (19). The ceRNA hypothesis states that non-coding RNAs like lncRNAs serve as microRNA (miRNA) sponges that competitively bind miRNA through miRNA response elements and inhibit miRNAs from binding to their target mRNAs and regulating their expression (16). The ceRNA network participates in carcinogenesis in various cancer types.

In the present study, we used high-throughput transcriptome sequencing on ivosidenib-treated NSCLC A549 and SK-MES-1 cells to identify differentially expressed RNAs. We performed a full-scale analysis of differentially expressed lncRNAs, miRNAs, and mRNAs using a bioinformatics approach. Finally, based on sequencing results, bioinformatics predictions, and ceRNA regulatory rules, we constructed a ceRNA network of lncRNAs, miRNAs, and mRNAs. Based on all of the above, for the first time, we elucidated the potential mechanisms of ivosidenib-mediated NSCLC cell suppression using transcriptome analysis. Our findings will help build a theoretical basis for future treatment of NSCLC using ivosidenib.

## Materials and Methods

### Cell Lines and Culture Conditions

We purchased five lung cancer cell lines (A549, NCI-H1650, NCI-H1299, SK-MES-1, NCI-H226) from the Cell Bank of the Chinese Academy of Sciences (Shanghai, China). A549, NCI-H1650, and NCI-H1299 are lung adenocarcinoma cell lines, and SK-MES-1 and NCI-H226 are lung squamous cell carcinoma cell lines. We obtained human bronchial epithelial cells (BEAS-2B) and dedicated culture solutions from the Cell Bank of the Chinese Academy of Sciences (Kunming, China). We maintained lung cancer cells in RPMI-1640 supplemented with 10% fetal bovine serum (FBS) and 100 U/ml penicillin/0.1 mg/ml streptomycin at 37°C with 5% CO_2_. We cultured BEAS-2B in a dedicated culture solution at 37°C with 5% CO_2_. We used A549 and SK-MES-1 that had relatively higher expression of IDH1 to perform functional experiments.

### Drug and Reagents

We purchased ivosidenib from MedChemExpress (Monmouth, NJ, USA). We dissolved ivosidenib powder in sterile dimethyl sulfoxide (DMSO) to prepare a 50 mM stock solution stored at −80°C. The 3-(4,5-dimethylthiazol-2-yl)-2,5-diphenyltetrazolium bromide (MTT) was from Sigma Chemical Corporation (St. Louis, MO, USA). We obtained the cell cycle detection kit from BD Biosciences (San Jose, CA, USA). TRIzol reagent was from Invitrogen (Carlsbad, CA, USA), RT reagent Kit and SYBR Green PCR Master Mix were from Promega (Madison, WI, USA). MiRNeasy Mini Kit, miRCURY LNA RT Kit, and miRCURY LNA SYBR Green PCR Kit were from QIAGEN (Valencia, CA, USA).

### RNA Extraction and RT-qPCR

We isolated total RNA from A549, NCI-H1650, NCI-H1299, SK-MES-1, NCI-H226, and BEAS-2B using TRIzol reagent and converted to cDNA according to the PrimeScript RT reagent kit manufacturer’s instructions. The cDNA underwent quantitative real-time PCR to detect IDH1, following the 2−ΔΔCT method analysis. We used B2M as an internal control. The primers sequences for the genes were as follows: IDH1, forward 5’-ACTGTAACCCGTCACTACCG; reverse 5’-AGTCCTTGGTCATGAAGCCA; B2M, forward 5’-AGCAGCATCATGGAGGTTTG; reverse 5’-AGCCCTCCTAGAGCTACCTG.

### Growth Inhibition Assay

We used the MTT assay to measured proliferation. We seeded cells in the log-phase in 96-well plates cultured overnight with five repeats for each group. We treated cells with various ivosidenib concentrations incubated for 24, 48, and 72 h at 37°C with 5% CO_2_. We then incubated the cells in MTT (0.25 mg/ml) for 4 h at 37°C. After medium removal, we lysed cells with DMSO. We measured absorbance 490 nm to determine the percentage of surviving cells.

### Colony-Formation Assay

We seeded A549 and SK-MES-1 cells in six-well plates with 200 and 500 cells per well, respectively. After adhering overnight, we treated the cells with various concentrations of ivosidenib for 2 weeks, and replaced medium every 3 days. To visualize the results, we fixed colonies in 4% paraformaldehyde and incubated them in crystal violet solution.

### Transwell Assay

Transwell migration assay occurred in chemotaxis chambers containing 24 wells. We inoculated cells into the upper chamber in 200 µl RPMI-1640 without serum that contained or did not contain ivosidenib. Bottom chambers contained RPMI-1640 medium containing 10% FBS. After 24 h of treatment, we fixed cells using 4% paraformaldehyde and stained with 0.25% crystal violet solution. The stained cells were counted using a microscope.

For the invasion assay, we added Matrigel (1:10 dilution) to the Transwell plate to form the matrix barrier. We resuspended cells in 200 µl contained 5% FBS RPMI 1640 medium that contained or did not contain ivosidenib and placed them in the upper chambers. We placed 600 µl 20% FBS RPMI 1640 medium in the lower chambers. After 48 h of treatment, we determined cell invasion using crystal violet staining. We imaged and counted stained cells as in the migration assay.

### Cell Cycle Assay

We incubated cells with various concentrations of ivosidenib for 24 h. We suspended cells in 70% ethanol, incubated them at 4°C overnight, and collected them using centrifugation at 1,500 rpm for 3 min. We then added 200 µl PI/Rnase A staining solution, and incubated cells for 60 min in the dark. We measured proportions of cells in each cycle using flow cytometry.

### Tumor Formation in BALB/c Nude Mice

We procured female BALB/c nude mice (5 weeks old) from the Beijing Charles River Laboratory Animal Technology Co., Ltd. (Beijing, China) to perform the xenograft experiments. We maintained all animals at 21–25°C, humidity 30–40%, and allowed them free access to food and water. To establish lung cancer xenograft model, we subcutaneously injected A549 cells (5 × 10^6^ cells) in the logarithmic phase of growth into the mice in their left flanks. We randomly subdivided mice into two groups: the control group (PBS, n = 4) and the drug-treated group (ivosidenib, n = 4). Subsequently, each mouse in the drug-treated group received once per day by oral gavage a dose of 150 mg/kg ivosidenib for 15 days (14). We measured tumor volumes every 3 days according to the following formula: V = (L×W^2^)/2, where L is the longer tumor diameter and W is the smaller diameter. We sacrificed the mice 24 h after the final dose, and isolated and weighed the subcutaneous tumors. We performed animal-related procedures according to the guide for the Care and Use of Laboratory Animals, with the approval of the Shanxi Medical University (Taiyuan, China).

### Whole-Transcriptome Sequencing

We performed RNA sequencing in drug-treated A549 cells (50 and 100 μM)/SK-MES-1 cells (75 and 100 μM) and their parent cell lines (A549 and SK-MES-1), Novogene Co., Ltd (Beijing, China).

### Bioinformatics Analysis

We considered genes with |log_2_FoldChange| >1 and adjusted *p*-values <0.05 as differentially expressed genes (DEGs). We considered DE-mRNAs, DE-miRNAs, and DE-lncRNAs with the same expression trend intersecting from 100 µM ivosidenib group of A549 and SK-MES-1 as common DE-mRNAs, DE-miRNAs, and DE-lncRNAs compared to control. We drew volcano maps to generate graphical overviews of expression profile using the ggplot2 package in R software (20). We used the heatmap package in R to plot the heat map of DE-RNAs (20). To explore the possible functions of DE-mRNAs, we performed gene ontology (GO) functional enrichment using DAVID (https://david.ncifcrf.gov/) and KEGG pathway enrichment analysis using KOBAS 3.0 (http://kobas.cbi.pku.edu.cn/kobas3) (20). Briefly, GO analyses consisted of three components: biological process (BP), cellular component (CC), and molecular function (MF). We considered *p <*0.05 Gene Ontology (GO) and Kyoto Encyclopedia of Genes and Genomes (KEGG) pathways as statistically significant. Then, we used the STRING online database (version 11.0 https://string-db.org/) to retrieve the protein-protein interactions (PPI). We visualized PPI pairs with a combined confidence score ≥0.4 in the network using Cytoscape 3.6.1 software (21).

### Construction of the LncRNA-miRNA-mRNA-Related ceRNA Regulatory Network

We screened target mRNAs of DE-miRNA using TargetScan (http://www.targetscan.org/). We predicted DE-miRNA target lncRNA using miRWalk2.0 (http://zmf.umm.uni-heidelberg.de/apps/zmf/mirwalk2/index.html). We selected miRWalk, TargetScan, and RNAhybrid to decode the relationships between the differentially expressed miRNAs and lncRNAs (22). According to the ceRNA regulatory mechanism and the changing trends of lncRNAs, miRNAs, and mRNAs, we constructed a ceRNA regulatory network using Cytoscape 3.6.1. Different shapes represent different RNA types, colors represent different regulated relationships.

### Validation of Significant miRNAs and Target Genes

We validated significant DE-ncRNAs and DE-mRNAs using quantitative reverse transcription-polymerase chain reaction (qRT-PCR). We obtained the TaqMan qRT‐PCR probes and primers for quantification of miRNAs from QIAGEN (Hilden, Germany) as follows: miR‐148a‐5p (product ID: YP00204188), miR-493-5p (product ID: YP00204166), and U6 (product ID: YP00203907). We used the 2−ΔΔCt method to calculate relative expression levels of mRNA, miRNA, and lncRNA, normalized to B2M or U6 snRNA. See **Table 1** for the display of the Gene primer list.

**Table 1:** The primers for qRT-PCR.

Name	Sequence (5’-3’)
DDIAS-Forward Primer	AGGTTCAGATGCCAGTAACTTCT
DDIAS-Reverse Primer	AGTGATTGTTAGGTGCCTGAGA
PLEKHO1-Forward Primer	AAACAGCCCGGTAACACGG
PLEKHO1-Reverse Primer	GGCATTGATCCACGATTCCTT
ZBED6-Forward Primer	GAAGGGTTTGCGAATTAAGGGG
ZBED6-Reverse Primer	GGGTCATTGGAAGCTAACAAAGC
SMAD5-Forward Primer	CCAGCAGTAAAGCGATTGTTGG
SMAD5-Reverse Primer	GGGGTAAGCCTTTTCTGTGAG
PCK2-Forward Primer	CATCCCAACTCTCGATTTTGTG
PCK2-Reverse Primer	TTCCCAGAAGTCCTTTGTGTTC
CHAC1-Forward Primer	GATTTTCGGGTACGGCTCCC
CHAC1-Reverse Primer	GAAGGTGTCTCCCTGCCAGA
PARD6G-AS1-Forward Primer	CCCACTGCCCTCCCTCCAAG
PARD6G-AS1-Reverse Primer	CGGTGTCTCCTGCTTTCTGTTCC
CTBP1-AS-Forward Primer	ACAACACAAAGCCCCGGAA
CTBP1-AS–Reverse Primer	GAAGAATGGTCTCGCCC
B2M-Forward Primer	AGCAGCATCATGGAGGTTTG
B2M-Reverse Primer	AGCCCTCCTAGAGCTACCTG

### Statistical Analysis

We used GraphPad Prism 6.0 software for all analyses. We analyzed cell functions and qRT-PCR outputs using continuous variable two-tailed Student’s t-tests. We analyzed sequencing data using bioinformatic tools. Statistical significance is presented in figures as *p < 0.05, **p < 0.01, or ***p < 0.001.

## Results

### IDH1 mRNA Expression in NSCLC Cell Lines

**Figure 1:**
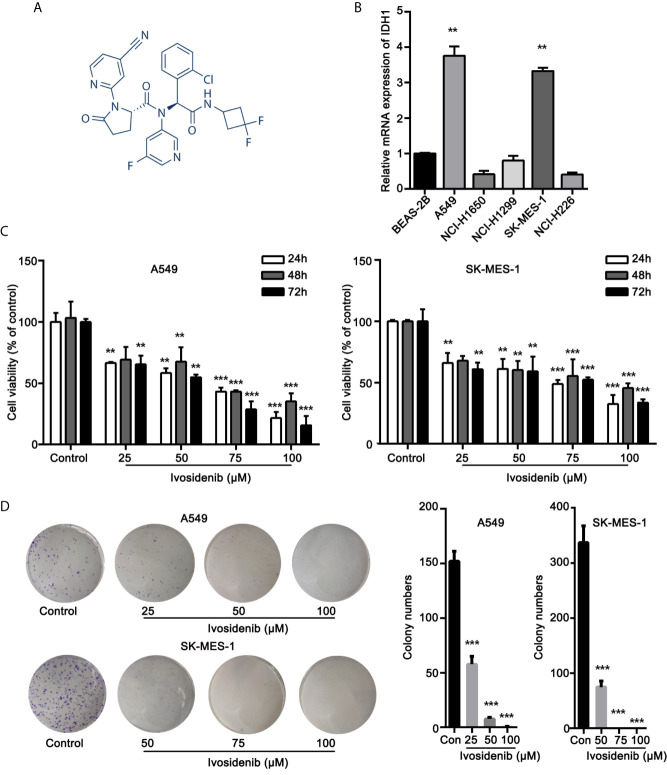
Ivosidenib inhibits the proliferation of NSCLC cells. **(A)** Chemical structure of ivosidenib. **(B)** IDH1 expression in NSCLC and BEAS-2B cell lines. **(C)** A549 and SK-MES-1 cells were treated with various concentrations of ivosidenib for 24, 48, and 72 h, and proliferation was measured using the MTT assay. Data are expressed as mean ± SD of three separate experiments. **(D)** Colony-formation assay (representative wells are presented). **p < 0.01, ***p < 0.001 *vs.* control.

The chemical structure of Ivosidenib is shown in **Figure 1A**. Ivosidenib may be a potential therapeutic drug candidate for NSCLC with high IDH1 expression. We measured IDH1 expression in five NSCLC cell lines (A549, NCI-H1299, NCI-H1650, SK-MES-1, and NCI-H226) and BEAS-2B. BEAS-2B, human normal bronchial epithelial cells, served as non-cancer reference lung cells. Expression levels of IDH1 were higher in A549 and SK-MES-1 cells than in normal BEAS-2B cells (**Figure 1B**).

### Ivosidenib Inhibits Proliferation, Migration, and Invasion of A549 and SK-MES-1 Cells

**Figure 2:**
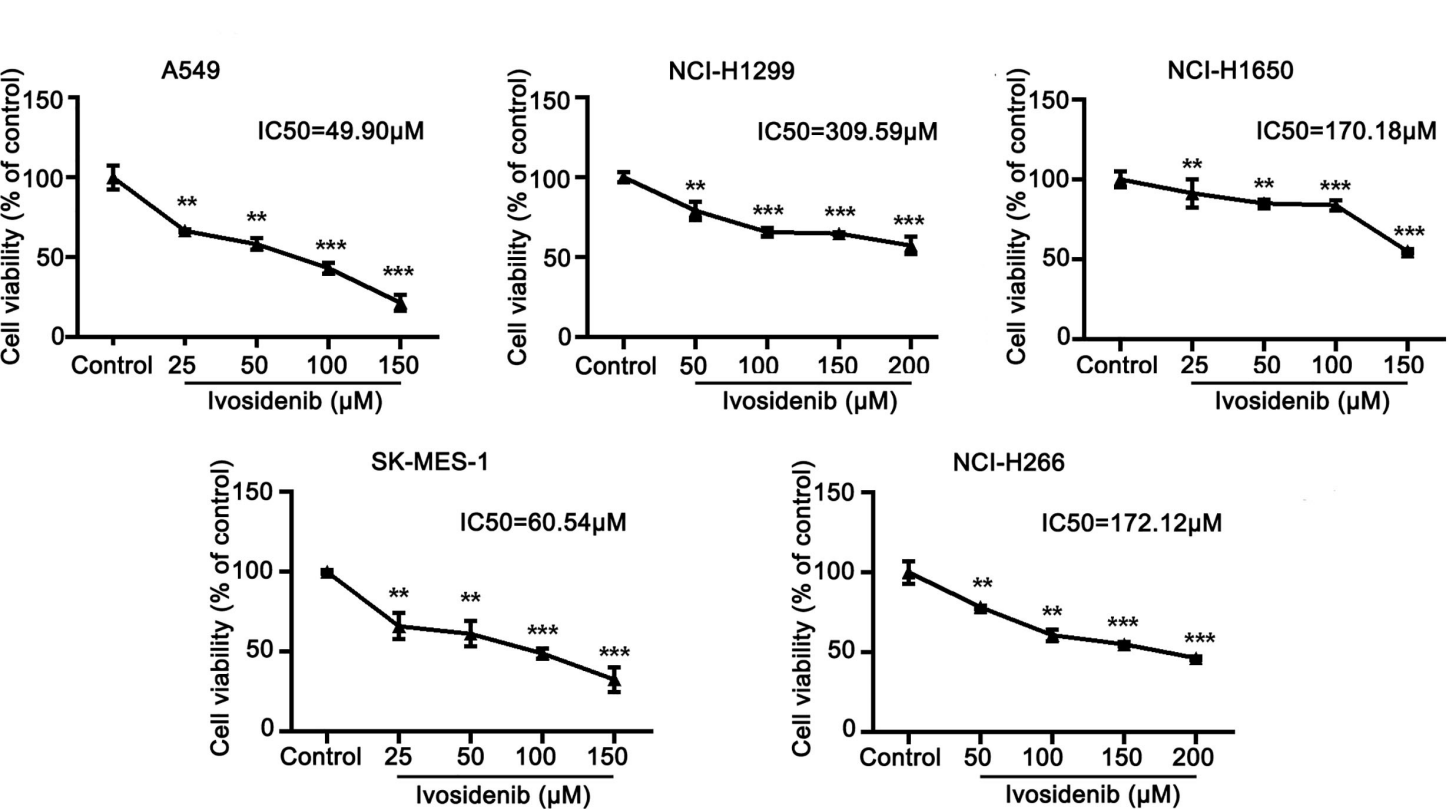
The sensitivity of different NSCLC cell lines to ivosidenib. NSCLC cells were treated with the control (RPMI-1640 culture) and various concentrations of ivosidenib for 24 h, cell proliferation was measured using a MTT assay. The MTT assay was performed to determine cell viability and values are expressed as the mean ± SD of three separate experiments. **p < 0.01, and ***p < 0.001 *vs.* Control.

**Figure 3:**
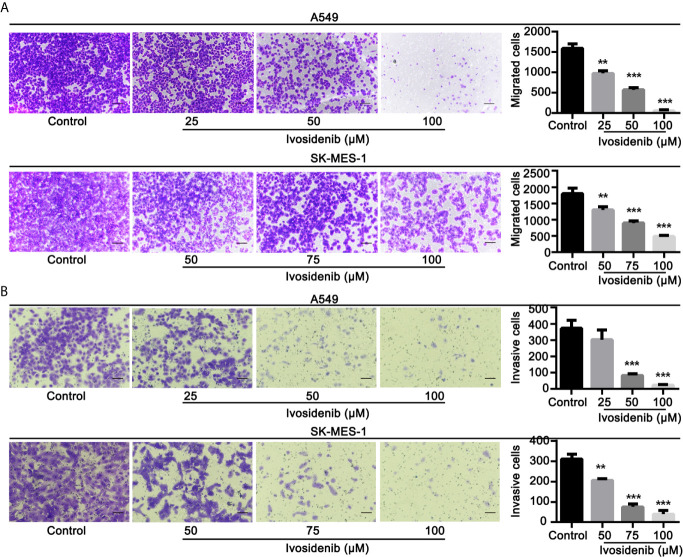
Ivosidenib inhibits invasion and migration of NSCLC cells. **(A)** A549 were treated with 25, 50, and 100 µM of ivosidenib for 24 h to measure migration. SK-MES-1 were treated with 50, 75, and 100 µM of ivosidenib for 24 h to measure migration. **(B)** A549 were treated with 25, 50, and 100 µM of ivosidenib for 48 h to measure migration. SK-MES-1 were treated with 50, 75, and 100 µM of ivosidenib for 48 h to measure migration. The quantitative results are shown in the right panel, and data are expressed as mean ± SD of three independent experiments. ** *p* < 0.01, and *** *p* < 0.001 *vs.* control.

To determine whether ivosidenib affects the biological behaviors of NSCLC cells, we conducted MTT assay, colony formation, and Transwell assay to measure the effects of ivosidenib on NSCLC cells. The NSCLC cell lines with high IDH1 expression were more sensitive to ivosidenib. The IC_50_ values of A549 and SK-MES-1 were 49.90 and 60.54 µM, respectively (**Figure 2**). A549 and SK-MES-1 cell viabilities were lower after treatment with ivosidenib for 24, 48, and 72 h. The suppression rates were dose-dependent but not time-dependent (**Figure 1C**). These findings suggest that 24 h of treatment was optimal, and therefore we chose this time course for subsequent experiments. As shown in (**Figure 1D**), the number of colonies inversely correlated with concentrations of ivosidenib. We performed a Transwell array in A549 and SK-MES-1 cells to evaluate the effect of ivosidenib on cell invasion and migration and found that the invasion and migration abilities of A549 and SK-MES-1 cells were significantly lower in the ivosidenib-treatment group than in the control group (**Figures 3A, B**).

### Ivosidenib Induces Cell Cycle Arrest in A549 and SK-MES-1 Cells

**Figure 4:**
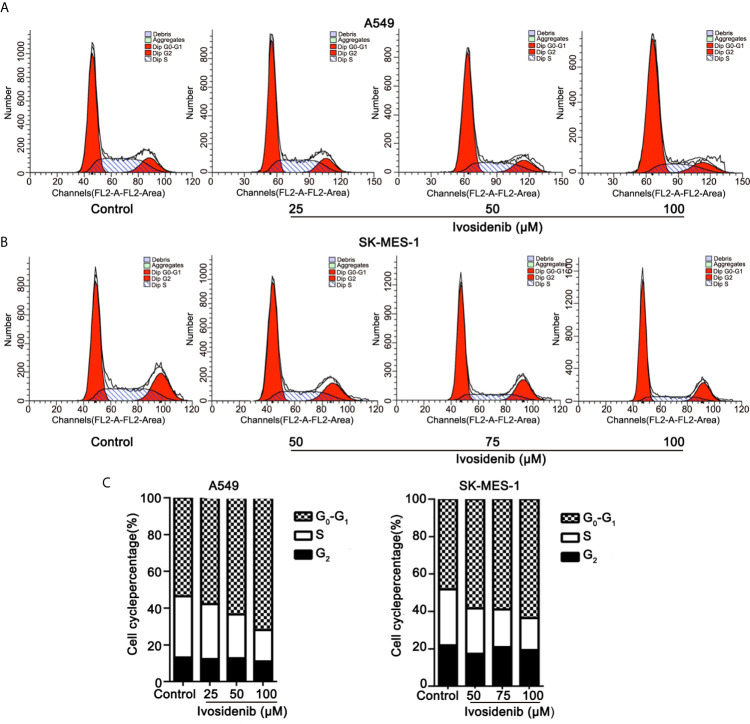
Ivosidenib inhibits the cell cycle in NSCLC cells. **(A)** Flow cytometric analysis was used to measure cell cycle distributions of A549 cells after 24 h of culture with ivosidenib at 25, 50, and 100 µM. **(B)** Flow cytometric analysis was used to measure cell cycle distributions of SK-MES-1 cells after 24 h of culture with ivosidenib at 50, 75, and 100 µM. **(C)** The results are reported in the diagram. Data represent the means ± SD of three separate experiments.

We measured the effects of ivosidenib on the cell cycle in A549 and SK-MES-1 cells using flow cytometry. Ivosidenib treatment gave rise to concentration-dependent cell cycle arrest at the G0-G1 phase (**Figure 4**). These findings suggest that ivosidenib efficiently suppresses the proliferation of A549 and SK-MES-1 cells.

### Ivosidenib Inhibits Tumor Growth in NSCLC Xenografted Mice

**Figure 5:**
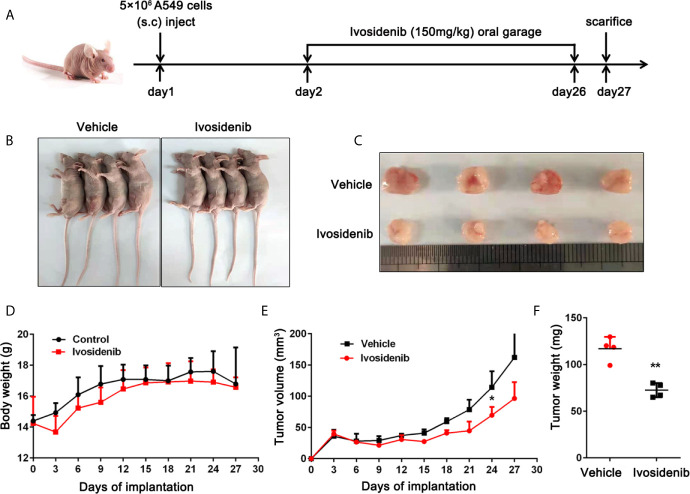
Ivosidenib inhibits the tumor growth *in vivo*. **(A)** Diagram of the animal study protocol. **(B)** Representative images show tumor xenografts. **(C)** The image and **(E)** weight of tumor harvested. **(F)** Tumor volumes and weight were measured, and the volume of the tumor was calculated [(length × width^2^)/2]. **(D)** Bodyweight calculated every 3 days after implantation. Data are expressed as the mean ± S.D. of five independent experiments. *p < 0.05; **p < 0.01 *vs.* the vehicle group.

To study the anti-tumor effect of ivosidenib *in vivo*, we established a xenograft nude mice model using A549 cells. The detailed experimental design is shown in (**Figure 5A**). As shown in **Figures 5B, C, E, F** compared with the vehicle group, the volumes and weights of the tumors were lower in the ivosidenib groups. Ivosidenib did not significantly affect body weights (**Figure 5D**), suggesting that the drug was not toxic at the experimental dose. These results suggest that ivosidenib inhibits tumor growth of NSCLC cells *in vivo*.

### Identification of Differentially Expressed mRNAs, miRNAs, and lncRNAs

**Figure 6:**
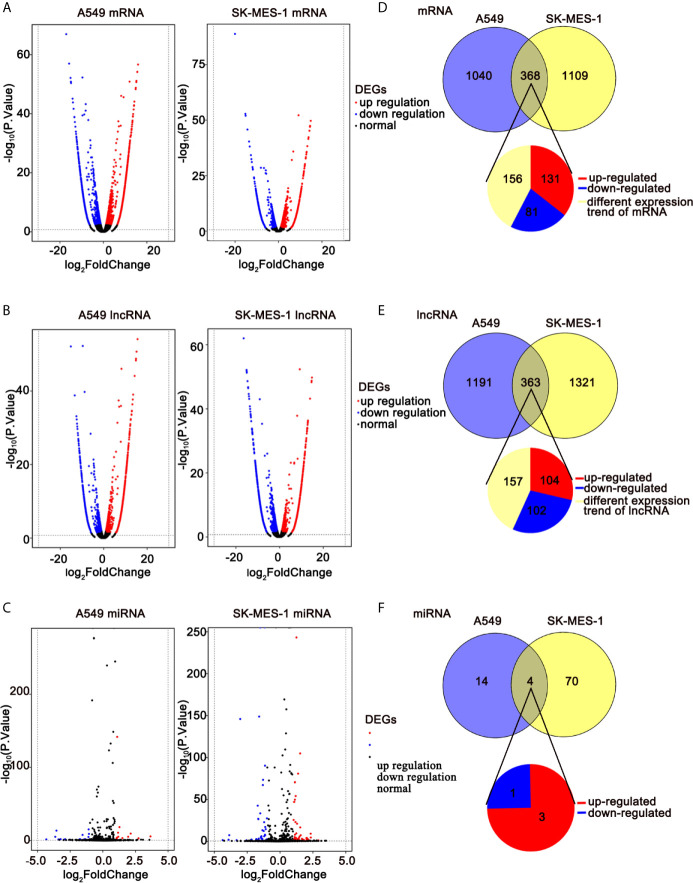
RNA-seq revealing distinct expression patterns of miRNAs, lncRNAs, and mRNAs in control of NSCLC cells and ivosidenib-treated NSCLC cells. **(A–C)** Volcano plot of DE-mRNAs, DE-lncRNAs, and DE-miRNA expression profiles between control NSCLC cells and ivosidenib-treated NSCLC cells. **(D–F)** Venn diagram showing the overlap number of DE-mRNA, DE-lncRNA, and DE-miRNA.

**Figure 7:**
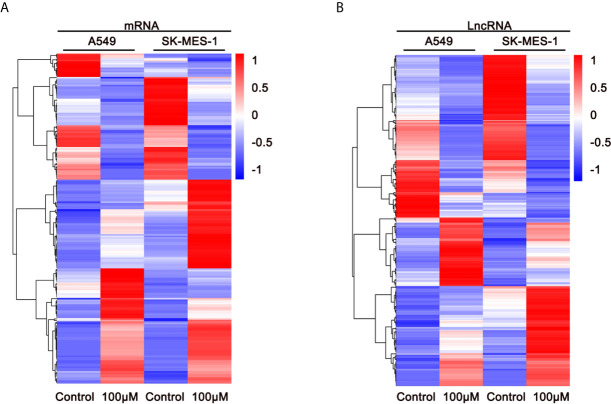
Expression profiles of DE-mRNAs are influenced by ivosidenib. Heatmap showing expression profiles of differentially expressed mRNAs **(A)** and lncRNAs **(B)** between NSCLC cells and ivosidenib-treated NSCLC cells.

To elucidate the anti-cancer mechanisms of ivosidenib, we performed RNA sequencing in drug-treated A549, SK-MES-1 cells, and their parent cell lines. Applying the cutoffs of *p*-value < 0.05 and |log2FoldChange| > 1, we selected DE-mRNAs. The volcano plot of mRNA revealed that a total of 1,408 and 1,477 mRNAs changed in the A549 and SK-MES-1, respectively (**Figure 6A**). Totals of 1,554 and 1,684 lncRNAs markedly changed in the A549 and SK-MES-1, respectively (**Figure 6B**). There were 18 altered miRNAs in A549 and 74 altered miRNAs in SK-MES-1 (**Figure 6C**). As shown in the Venn diagram, 212 DE-mRNAs (131 up- and 81 down-regulated), 206 DE-lncRNAs (104 up- and 102 down-regulated), and 4 DE-miRNAs (three up- and one down-regulated) appeared from two cells (**Figures 6D–F**). Heatmap analysis visually displayed expression levels of DE-mRNAs and DE-lncRNAs (**Figures 7A, B**).

### Functional Enrichment Analysis of DE-mRNAs

**Figure 8:**
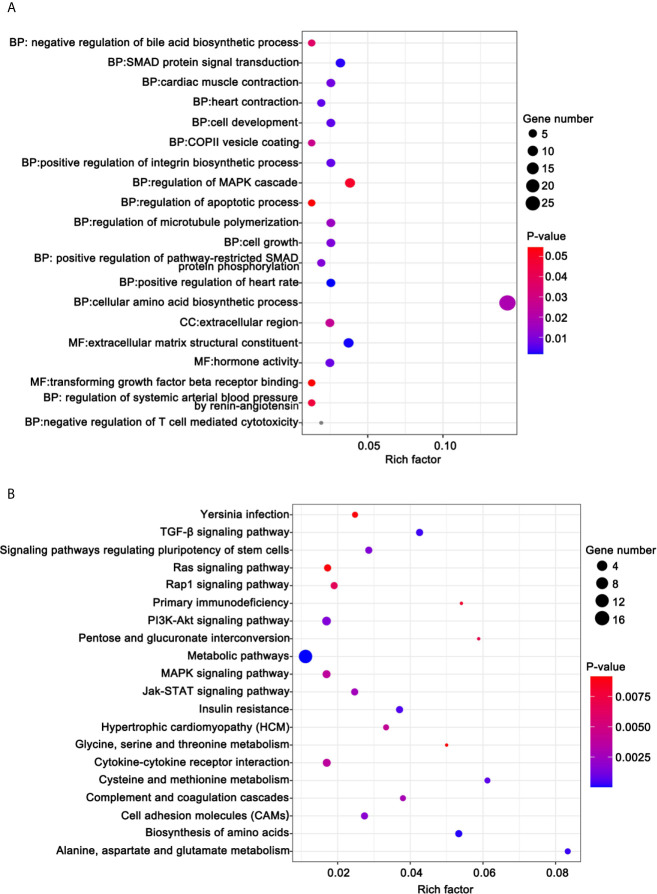
Functional analysis for the DE-mRNAs. **(A)** Bubble diagram of the top 20 ranked GO terms of DE-mRNAs. The vertical axis indicates GO terms and the horizontal axis represents the Rich factor. The enrichment degree was stronger with a larger Rich factor. Size of the dots indicates the number of genes in the GO term. **(B)** KEGG pathway enrichment analysis of CDE-mRNAs. The vertical axis indicates the different pathways. The enrichment degree was stronger with a higher enrichment ratio. The horizontal axis indicates the Rich factor. The enrichment degree was stronger with a larger Rich factor. Size of the dots indicates the number of genes in the pathways.

To illuminate the biological functions of DE-mRNAs, we performed GO term enrichment analysis and KEGG pathway analysis. **Figure 8A** shows the top 20 ranked GO in terms of DE-mRNAs. DE-mRNAs ontology (GO) enrichment analysis revealed that the majority of these genes are enriched in cellular amino acid biosynthetic processes, SMAD protein signal transduction, regulation of the MAPK cascade, transforming growth factor β receptor binding, regulation of apoptosis, and others. GO enrichment analysis indicated that these cell processes were the most influential processes affected by ivosidenib.

KEGG pathway analysis helps us better understand the biological function of genes. We screened 41 pathways with significantly differential expressions (*p <*0.05) (**Table S1**). **Figure 8B** shows the top 20 ranked significant pathways in KEGG. DE-mRNAs were significantly enriched in metabolic and cancer-associated pathways including metabolic pathways, biosynthesis of amino acids, alanine, aspartate, and glutamate metabolism, the PI3K-Akt signaling pathway, the TGF-β signaling pathway, cell adhesion molecules (CAMs), the Jak-STAT signaling pathway, the MAPK signaling pathway, and the Rap1 signaling pathway.

**Figure 9:**
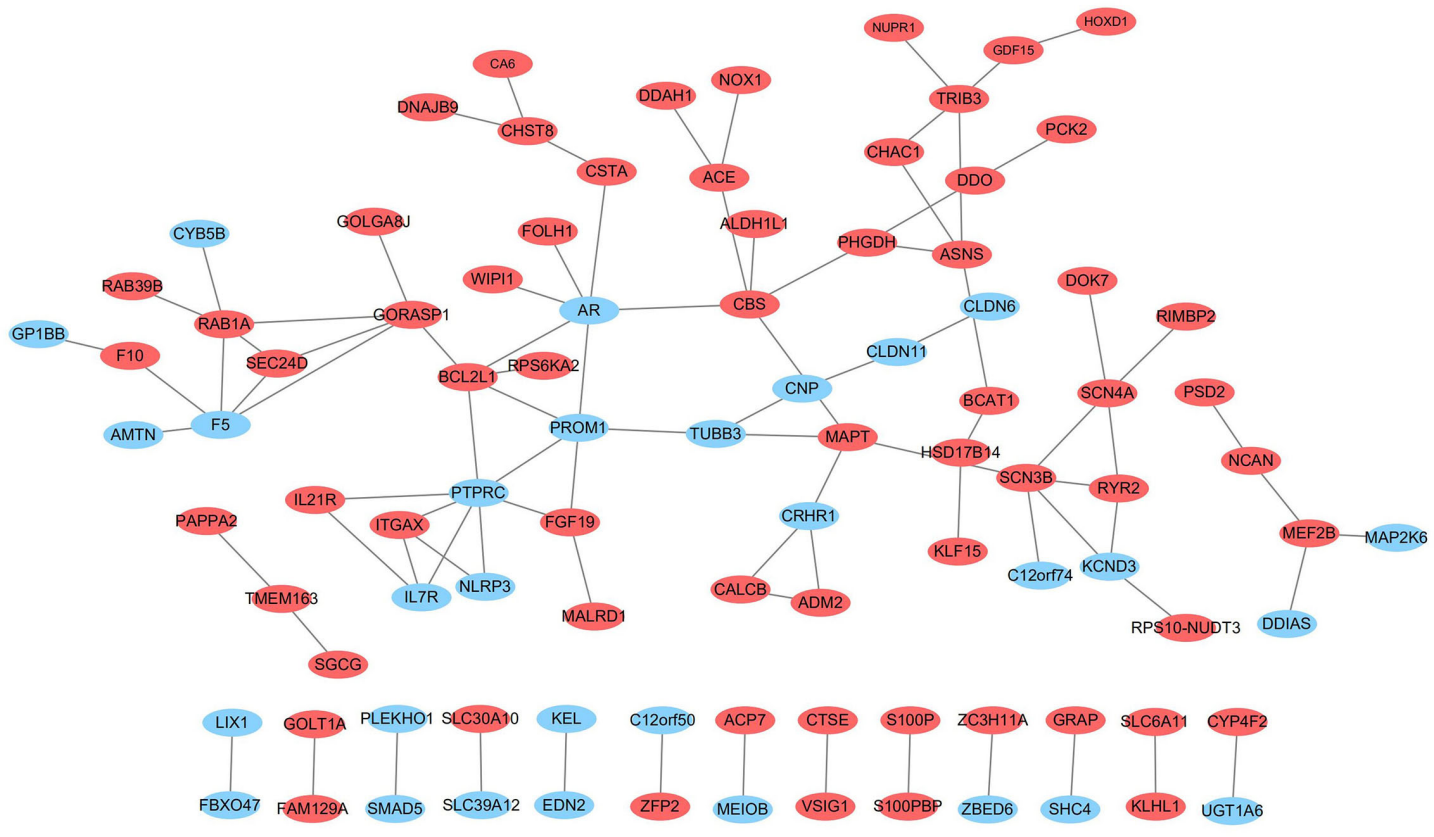
The network of protein-protein interactions (PPI) of differentially expressed genes. Red and blue represent up- and down-regulation, respectively.

To explore the connections of these DE-mRNAs, we established a protein-protein interaction (PPI) network using the STRING online database. We used Cytoscape (version 3.6.1) to visualize the PPI network (**Figure 9**). The results may provide important information regarding the activity of ivosidenib in NSCLC A549 and SK-MES-1 cells.

### Validation of DE-mRNA and DE-ncRNA Expression

**Figure 10:**
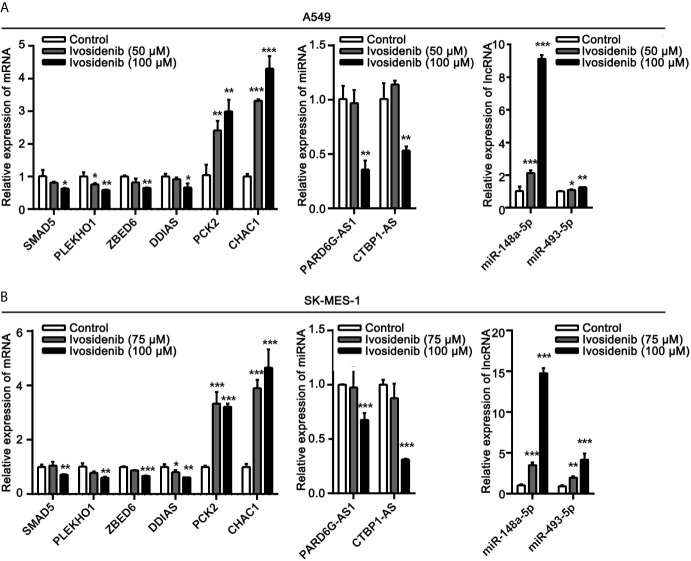
QRT-PCR analysis of the expressions of DE-RNAs. **(A)** After treating with ivosidenib for 24 h, DE-RNAs expression was determined using RT-qPCR in A549 cells. **(B)** After treating with ivosidenib for 24 h, DE-RNAs expression was determined using RT-qPCR in SK-MES-1 cells. *p < 0.05, **p < 0.01, and ***p < 0.001.

To determine the accuracy of transcriptome sequencing, we randomly selected 10 DE-RNAs to verify the reliability of high-throughput RNA sequencing using qRT-PCR. As shown in **Figure 10**, the agreement between their expression trends and RNA-seq data reflects the reliability of RNA-seq data. According to the RNA-seq and qRT-PCR results, expressions levels of four mRNAs (SMAD5, PLEKHO1, ZBED6, DDIAS) and two lncRNAs (PARD6G-AS1, CTBP1-AS) were lower after ivosidenib treatment; expression levels of two mRNAs (CHAC1, PCK2) and two miRNAs (miR-148a-5p, miR-493-5p) were greater after ivosidenib treatment (**Figure 10**).

### Construction of a ceRNA Regulatory Network

**Figure 11:**
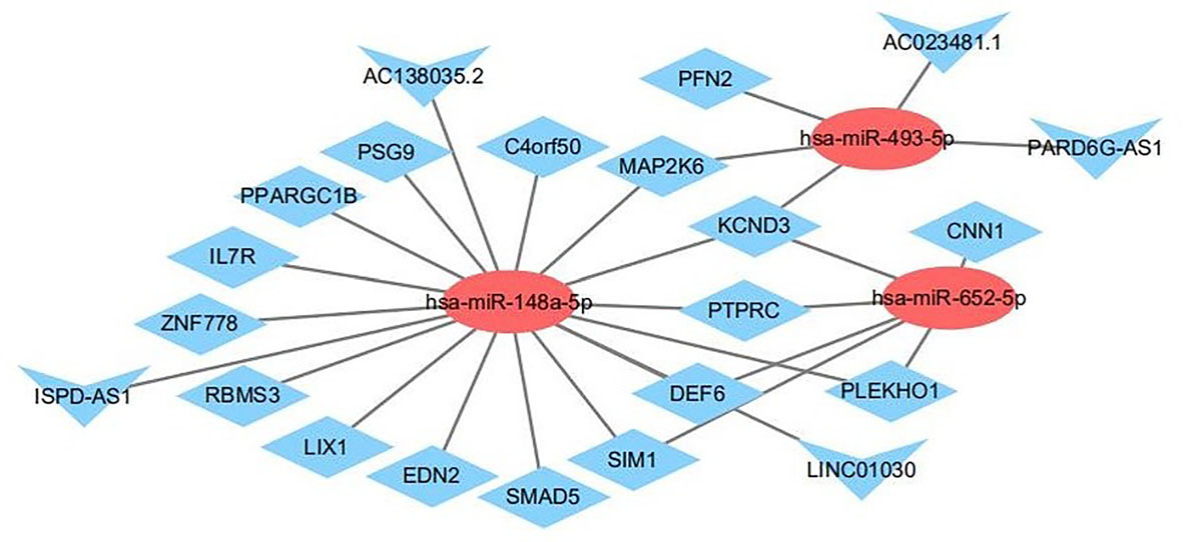
The interaction network of lncRNA–miRNA–mRNA. Red and blue represent up- and down-regulation, respectively. Triangles represent DE-lncRNAs, circles represent DE-miRNAs, and diamonds represent DE-mRNAs.

To further explore the roles of the altered DE-lncRNAs, DE-miRNAs, and DE-mRNAs in drug-treated NSCLC cell lines and to clarify the relationships among them, we generated a ceRNA regulatory network. First, we used Targetscan to decode the relationships between the altered miRNAs and mRNAs. The algorithm predicted three miRNAs to interact with 68 DEmRNAs (**Table S2**). Next, we used miRWalk, TargetScan, and RNAhybrid to analyze the relationships between the altered lncRNAs and miRNAs (**Table S3**). We selected the miRNAs that were negatively regulated by the lncRNAs and mRNAs to build the LncRNA-miRNA-mRNA network. We used Cytoscape (version 3.6.1) to visualize the ceRNA network. We constructed a ceRNA network including three DE-miRNAs (miR-148a-5p, miR-652-5p, and miR-493-5p), 17 target DE-mRNAs (SMAD5, PLEKHO1, PFN2, IL7R, ZNF778, MAP2K6, and others), and five target DE-lncRNAs (PARD6G-AS1, ISPD-AS1, LINC01030, AC023481.1, and AC138035.2) (**Figure 11**).

To further understand the potential function of mRNAs and to provide useful information for experiments, we identified a lncRNA-miRNA-mRNA axis based on the results of the functional analysis. From the previous steps, we identified several GO terms and KEGG pathways. Next, we reorganized the mRNAs that significantly correlated with cancer progression and linked their upstream miRNA and lncRNA, such as ISPD-AS1-has-miR-148a-5p-SMAD5, and PARD6G-AS1-has-miR-493-5p-PFN2 (**Table 2**). We believe these axes will provide more information and that are appropriate for experiments. In the future, much more lab experiments need to be conducted to further validate these findings.

**Table 2:** Cancer-Related lncRNA-miRNA-mRNA Axis.

mRNA	miRNA	lncRNA	Function Term
SMAD5	hsa-miR-148a-5p	AC138035.2	TGF-β signaling pathway
		ISPD-AS1	
		LINC01030	
IL7R	hsa-miR-148a-5p	AC138035.2	PI3K-Akt signaling pathway
		ISPD-AS1	Jak-STAT signaling pathway
		LINC01030	
MAP2K6	hsa-miR-148a-5p	AC138035.2	MAPK signaling pathway
		ISPD-AS1	Rap1 signaling pathway
		LINC01030	
	hsa-miR-493-5p	PARD6G-AS1	
		AC023481.1	
PFN2	hsa-miR-493-5p	PARD6G-AS1	Rap1 signaling pathway
		AC023481.1	
PTPRC	hsa-miR-148a-5p	AC138035.2	Cell adhesion molecules (CAMs)
		ISPD-AS1	
		LINC01030	

## Discussion

The US Food and Drug Administration approved the use of ivosidenib, an inhibitor of IDH1, to treat acute myelogenous leukemia (AML) harboring IDH1 mutations in a phase I clinical trial (23). In the present study, levels of proliferation, migration, and invasion of NSCLC cells (A549, SK-MES-1) were significantly lower, and cell cycle arrested at the G0-G1 phase after ivosidenib treatment. Based on this, it appears that ivosidenib acts by inhibiting cell proliferation but not by inducing cell death. The drug efficiently inhibits NSCLC progression *in vivo*. A previous study showed that knockdown of IDH1 by RNA interference reduced the proliferative capacity of NSCLC cells and significantly decreased the growth of xenograft tumors *in vivo* (11). Our results indicated that, as a mIDH1 inhibitor, ivosidenib might be appropriate for the treatment of NSCLC even without IDH1 mutation.

Using whole transcriptome resequencing, we identified 212 DE-mRNAs, 4 DE-miRNAs, and 206 DE-lncRNAs and analyzed their function and KEGG pathway, as well as the connections between mRNA, lncRNA, and miRNA. We selected the lncRNA-miRNA-mRNA axis according to the results of functional analysis. In the top 20 KEGG pathways, we identified cancer-related pathways, including the TGF-β signaling pathway (involving SMAD5), the PI3K-Akt signaling pathway (involving IL7R), the Jak-STAT signaling pathway (involving IL7R), the MAPK signaling pathway (involving genes MAP2K6), the Rap1 signaling pathway (involving MAP2K6 and PFN2), and cell adhesion molecules (involving PTPRC) (**Table 2**). Pioneering studies demonstrated that alterations of these genes might result in tumorigenesis and development; these include SMAD5 (24, 25), IL7R (26), MAP2K6 (27), and PFN2 (28, 29).

Subsequently, we evaluated the effects of ivosidenib on ncRNAs. Ivosidenib induced the up-regulation of miR-493-5p and miR-148a-5p. Other studies reported the tumor-inhibiting activity of miR-493-5p in malignant tumors (30–32). Studies showed that overexpression of miR-493-5p suppressed NSCLC growth, migration, and invasion (30). MiR-148a-5p associates with NSCLC progression (33, 34). Zhang et al. suggested that miR-148a-5p suppresses proliferation and migration and induces apoptosis in NSCLC cells *via* the PI3K/AKT signaling pathway by targeting ERBB3 and ITGA5 (34). We found that expression levels of miR-493-5p and miR-148a-5p were higher in ivosidenib-treated NSCLC cells. These findings suggest that miR-493-5p and miR-148a-5p may participate in the anti-NSCLC mechanism of ivosidenib.

Several lines of evidence suggest that lncRNAs interfere with miRNA activity as endogenous sponges. In the present study, based on the constructed lncRNA–miRNA–mRNA network, we observed that many lncRNAs contained one or more miRNA binding sites. LncRNAs (AC138035.2, ISPD-AS1, LINC01030, PARD6G-AS1, and AC023481.1) interacted with SMAD5, IL7R, MAP2K6, and PFN2, through competitively binding with miR-493-5p or miR-148a-5p. Further study may reveal the interaction relationships of lncRNA–miRNA–mRNA in the mechanism of action of ivosidenib (**Table 2**).

In summary, ivosidenib significantly inhibited the proliferation, migration, and invasion of NSCLC cells, a result of interactions among multiple pathways and signal molecules. Interfering with a series of signal pathways, including TGF-β, PI3K-Akt, Jak-STAT, MAPK, Rap1, and cell adhesion molecules, ivosidenib influenced the malignant phenotype of NSCLC cells. Our findings helped elucidate the potential mechanism of ivosidenib, building a regulatory ceRNA in NSCLC cells, and laying the foundation for further experimental and clinical studies of ivosidenib.

## Data Availability Statement

The datasets presented in this study can be found in online repositories. The names of the repository/repositories and accession number(s) can be found below: NCBI database, and the SRA accession number is SRR13363999, SRR13364000, SRR13364001, SRR13363997, SRR13363996, SRR13363995, SRR13363994 and SRR13363998. The bioproject number is PRJNA689881.

## Ethics Statement

The animal study was reviewed and approved by Shanxi Medical University.

## Author Contributions

JW and RC performed the experiments, collected and analyzed the data, and took part in writing the manuscript. HS, TY, YQ, YZ, and ZH performed the research and collected the data. PK, MP, and XZ initiated, designed the study, analyzed the data, and drafted the paper. All authors contributed to the article approved the submitted version.

## Funding

This study was funded by The Special Fund Project for Guiding Local Science and Technology Development by the Central Government (No.: YDZX20191400002737).

## Conflict of Interest

The authors declare that the research was conducted in the absence of any commercial or financial relationships that could be construed as a potential conflict of interest.
