# Supplementary material for: Transcriptome Analysis of Ivosidenib-Mediated Inhibitory Functions on Non-Small Cell Lung Cancer
Source: Front Oncol. 2021 Mar 30;11:626605. doi: 10.3389/fonc.2021.626605 (PMC8042334; doi:10.3389/fonc.2021.626605)
Supplement: Supplementary file 1 [file Table_1.doc]

**Table S1 The KEGG pathway**

| Term | P-Value | Input | |
| --- | --- | --- | --- |
| Metabolic pathways  Biosynthesis of amino acids  Alanine, aspartate and glutamate metabolism  TGF-beta signaling pathway  Insulin resistance  Cysteine and methionine metabolism  Signaling pathways regulating pluripotency of stem cells  PI3K-Akt signaling pathway  Cell adhesion molecules (CAMs)  Jak-STAT signaling pathway  Complement and coagulation cascades  Cytokine-cytokine receptor interaction  MAPK signaling pathway  Hypertrophic cardiomyopathy  Rap1 signaling pathway  Pentose and glucuronate interconversions  Primary immunodeficiency  Yersinia infection  Ras signaling pathway  Glycine, serine and threonine metabolism  Vascular smooth muscle contraction  Fluid shear stress and atherosclerosis  Amyotrophic lateral sclerosis (ALS)  Pathogenic Escherichia coli infection  Glycerolipid metabolism  NOD-like receptor signaling pathway  Renin secretion  Transcriptional misregulation in cancer  Chronic myeloid leukemia  Arrhythmogenic right ventricular cardiomyopathy (ARVC)  Synaptic vesicle cycle  EGFR tyrosine kinase inhibitor resistance  ECM-receptor interaction  Pathways in cancer  Human immunodeficiency virus 1 infection  Gap junction  Regulation of actin cytoskeleton  Small cell lung cancer  Dilated cardiomyopathy (DCM)  Hematopoietic cell lineage  AGE-RAGE signaling pathway in diabetic complications | 4.30E-05  0.000161971  0.000327038  0.000370245  0.00061339  0.000765483  0.00155893  0.0015671  0.001809671  0.002612455  0.002845936  0.003780557  0.003834204  0.004057434  0.006413275  0.006768365  0.00790772  0.008978742  0.008978904  0.009127009  0.011298115  0.012937354  0.014253897  0.016362737  0.019757931  0.024454774  0.024698413  0.027322009  0.029388527  0.030085363  0.030788745  0.031498621  0.036645234  0.037309966  0.037819559  0.038171216  0.038700935  0.042090061  0.044510656  0.04532878  0.047816272 | | PCK2|CHAC1|PHGDH|CBS|ADA2|ASNS|BCAT1|ACSM4|CHST8|AR|DDO|FUT1|UGT1A6|CA6|CYP4F2|FOLH1  CBS|BCAT1|ASNS|PHGDH  DDO|ASNS|FOLH1  SMAD5|INHBC|INHBE|LEFTY1  PCK2|RPS6KA2|PPARGC1B|TRIB3  CBS|BCAT1|PHGDH  SMAD5|INHBC|INHBE|LEFTY1  BCL2L1|PCK2|IL7R|FGF19|COL4A3|ANGPT4  LRRC4C|CLDN11|PTPRC|CLDN6  BCL2L1|FHL1|IL7R|IL21R  ITGAX|F10|F5  IL7R|INHBC|GDF15|IL21R|INHBE  RPS6KA2|FGF19|MAP2K6|MAPT|ANGPT4  SGCG|RYR2|ACE  PFN2|FGF19|MAP2K6|ANGPT4  UGT1A6|AR  IL7R|PTPRC  RPS6KA2|MAP2K6|NLRP3  BCL2L1|SHC4|FGF19|ANGPT4  CBS|PHGDH  CALCB|EDN2|CNP  NOX1|MAP2K6|CNP  BCL2L1|MAP2K6  TUBB3|TUBB8  AR|PLPP5  BCL2L1|TRPC7|NLRP3  EDN2|ACE  BCL2L1|NUPR1|PROM1  BCL2L1|SHC4  SGCG|RYR2  SLC6A11|SLC17A8  BCL2L1|SHC4  COL4A3|GP1BB  BCL2L1|HLF|FGF19|COL4A3|IL7R  BCL2L1|WEE2|MAP2K6  TUBB3|TUBB8  ITGAX|PFN2|FGF19  BCL2L1|COL4A3  SGCG|RYR2  IL7R|GP1BB  NOX1|COL4A3 |
