# Supplementary material for: Transcriptome Analysis of Ivosidenib-Mediated Inhibitory Functions on Non-Small Cell Lung Cancer
Source: Front Oncol. 2021 Mar 30;11:626605. doi: 10.3389/fonc.2021.626605 (PMC8042334; doi:10.3389/fonc.2021.626605)
Supplement: Supplementary file 2 [file Table_2.doc]

**Table S2 The list of miRNA-mRNA pair**

| miRNA | miRNA regulation | mRNA | mRNA regulaion |
| --- | --- | --- | --- |
| hsa-miR-148a-5p  hsa-miR-148a-5p  hsa-miR-148a-5p  hsa-miR-148a-5p  hsa-miR-148a-5p  hsa-miR-148a-5p  hsa-miR-148a-5p  hsa-miR-148a-5p  hsa-miR-148a-5p  hsa-miR-148a-5p  hsa-miR-148a-5p  hsa-miR-148a-5p  hsa-miR-148a-5p  hsa-miR-148a-5p  hsa-miR-148a-5p  hsa-miR-148a-5p  hsa-miR-148a-5p  hsa-miR-148a-5p  hsa-miR-148a-5p  hsa-miR-148a-5p  hsa-miR-148a-5p  hsa-miR-148a-5p  hsa-miR-148a-5p  hsa-miR-148a-5p  hsa-miR-148a-5p  hsa-miR-148a-5p  hsa-miR-148a-5p  hsa-miR-148a-5p  hsa-miR-148a-5p  hsa-miR-148a-5p  hsa-miR-148a-5p  hsa-miR-148a-5p  hsa-miR-148a-5p  hsa-miR-148a-5p  hsa-miR-148a-5p  hsa-miR-148a-5p  hsa-miR-148a-5p  hsa-miR-148a-5p  hsa-miR-148a-5p  hsa-miR-493-5p  hsa-miR-493-5p  hsa-miR-493-5p  hsa-miR-493-5p  hsa-miR-493-5p  hsa-miR-493-5p  hsa-miR-493-5p  hsa-miR-493-5p  hsa-miR-652-5p  hsa-miR-652-5p  hsa-miR-652-5p  hsa-miR-652-5p  hsa-miR-652-5p  hsa-miR-652-5p  hsa-miR-652-5p  hsa-miR-652-5p  hsa-miR-652-5p  hsa-miR-652-5p  hsa-miR-652-5p  hsa-miR-652-5p  hsa-miR-652-5p  hsa-miR-652-5p  hsa-miR-652-5p  hsa-miR-652-5p  hsa-miR-652-5p  hsa-miR-652-5p  hsa-miR-652-5p  hsa-miR-652-5p  hsa-miR-652-5p | UP  UP  UP  UP  UP  UP  UP  UP  UP  UP  UP  UP  UP  UP  UP  UP  UP  UP  UP  UP  UP  UP  UP  UP  UP  UP  UP  UP  UP  UP  UP  UP  UP  UP  UP  UP  UP  UP  UP  UP  UP  UP  UP  UP  UP  UP  UP  UP  UP  UP  UP  UP  UP  UP  UP  UP  UP  UP  UP  UP  UP  UP  UP  UP  UP  UP  UP  UP | ULBP1  EDN2  CALCB  MDFIC  SMAD5  PSG9  LIX1  EFCC1  IL7R  PTPRC  KCND3  KLHL1  SLFN5  FHL1  SLC30A10  SCN3B  COL4A3  RPS6KA2  SEC24D  FAM129A  S100PBP  SIM1  MAP2K6  SLC17A8  C4orf50  DEF6  PLEKHO1  RBMS3  KLF15  FUT1  PPARGC1B  CTSE  ZNF778  ZC3H11A  RNF125  ZNF423  RAB1A  PLGLB2  BCAT1  PFN2  PSD2  FGF19  VSIG1  RNF125  AFF3  KCND3  MAP2K6  DAZAP2  ACSM4  CNN1  BCAT1  INHBE  PTPRC  FAM129A  SIM1  MAPT  LURAP1  NCAN  NCMAP  KCND3  AGR2  DEF6  PAPPA2  PLEKHO1  MDFIC  ZNF423  TG  F10 | UP  DOWN  UP  UP  DOWN  DOWN  DOWN  UP  DOWN  DOWN  DOWN  UP  UP  UP  UP  UP  UP  UP  UP  UP  UP  DOWN  DOWN  UP  DOWN  DOWN  DOWN  DOWN  UP  UP  DOWN  UP  DOWN  UP  UP  UP  UP  UP  UP  DOWN  UP  UP  UP  UP  UP  DOWN  DOWN  UP  UP  DOWN  UP  UP  DOWN  UP  DOWN  UP  UP  UP  UP  DOWN  UP  DOWN  UP  DOWN  UP  UP  UP  UP |
