# Supplementary material for: Transcriptome Analysis of Ivosidenib-Mediated Inhibitory Functions on Non-Small Cell Lung Cancer
Source: Front Oncol. 2021 Mar 30;11:626605. doi: 10.3389/fonc.2021.626605 (PMC8042334; doi:10.3389/fonc.2021.626605)
Supplement: Supplementary file 3 [file Table_3.doc]

**Table S3 The list of miRNA-lncRNA pair**

| miRNA | miRNA regulation | lncRNA | lncRNA regulaion |
| --- | --- | --- | --- |
| hsa-miR-148a-5p  hsa-miR-148a-5p  hsa-miR-148a-5p  hsa-miR-148a-5p  hsa-miR-148a-5p  hsa-miR-148a-5p  hsa-miR-148a-5p  hsa-miR-148a-5p  hsa-miR-493-5p  hsa-miR-493-5p  hsa-miR-493-5p  hsa-miR-652-5p | UP  UP  UP  UP  UP  UP  UP  UP  UP  UP  UP  UP | LINC00501  AC138035.2  LINC00504  ISPD-AS1  LINC01030  AF038458.3  TTC39A-AS1  HYMAI  PARD6G-AS1  HYMAI  AC023481.1  LINC00471 | UP  DOWN  UP  DOWN  DOWN  UP  UP  UP  DOWN  UP  DOWN  UP |
